# Refining Prognostic Stratification in Clear Cell Renal Cell Carcinoma: Genomic, Tissue-Based, Circulating Biomarkers and Integrated Models

**DOI:** 10.3390/cancers18091371

**Published:** 2026-04-25

**Authors:** Mariana Bianca Chifu, Simona Eliza Giușcă, Andrei Daniel Timofte, Constantin Aleodor Costin, Andreea Rusu, Ana-Maria Ipatov, Irina Draga Căruntu

**Affiliations:** 1 Department of Morfofunctional Sciences 1, “Grigore T. Popa” University of Medicine and Pharmacy, 700115 Iași, Romania; bianca.manole@umfiasi.ro (M.B.C.); andrei-daniel.timofte@umfiasi.ro (A.D.T.); aleodor.costin@umfiasi.ro (C.A.C.); andreea-rusu@umfiasi.ro (A.R.); irina.caruntu@umfiasi.ro (I.D.C.); 2 Department of Pathology, “Dr. C. I. Parhon” University Hospital, 700503 Iași, Romania; 3 Department of Preventive Medicine and Interdisciplinarity, “Grigore T. Popa” University of Medicine and Pharmacy, 700115 Iași, Romania; ipatovanamaria@gmail.com; 4 Romanian Medical Science Academy, 030171 Bucharest, Romania

**Keywords:** clear cell renal cell carcinoma, prognostic biomarkers, genomic alterations, tissue-based biomarkers, circulating biomarkers, multi-omic integration

## Abstract

Clear cell renal cell carcinoma (ccRCC) is characterized by marked biological heterogeneity, which limits the prognostic accuracy of conventional clinicopathological models. Increasing attention has therefore focused on identification of biomarkers that can enhance risk stratification throughout all stages of the disease. Starting from the current state of the art, this narrative review summarizes and critically appraises the evidence published over the past decade regarding prognostic biomarkers in ccRCC. The analysis is structured into four overarching domains: (i) genomic biomarkers, covering somatic alterations and transcriptomic signatures; (ii) tissue-based biomarkers, including immunohistochemical surrogates and immune microenvironment features; (iii) circulating biomarkers, such as systemic inflammation parameters and indices; and (iv) integrated predictive models, represented by emerging multi-omic approaches. Going through the broad framework of potential prognostic biomarkers, emphasis is placed on their individual and integrative value in relation to classic clinical-pathological factors and survival parameters. At the tissue level, chromosome 3p-related alterations constitute a central molecular feature of ccRCC. Among these, *BAP1* loss has emerged as one of the most consistently validated indicators of aggressive tumor behavior. Disruption of the *SETD2*/H3K36me3 axis and immune-related biomarkers, including PD-L1 expression, have demonstrated prognostic associations in selected settings, although with variable and context-dependent performance. In the circulating compartment, plasma KIM-1 has shown prognostic relevance following nephrectomy, while postoperative detection of circulating tumor DNA (ctDNA) may identify patients at increased risk of recurrence. However, limited analytical sensitivity and methodological heterogeneity currently restrict the broader clinical applicability of ctDNA-based strategies. Systemic inflammatory indices, such as the neutrophil-to-lymphocyte ratio, show reproducible associations with outcomes but largely reflect host inflammatory status rather than tumor-specific biology. However, no single biomarker currently supports routine prognostic implementation in ccRCC. Future progress will likely depend on integrative models combining genomic, tissue-based, immune, and circulating parameters with established clinical variables. Prospective validation and clear demonstration of incremental clinical utility will be essential before such strategies can meaningfully inform therapeutic decision-making.

## Simple Summary

Kidney cancer is a complex disease in which patients with similar clinical features can experience very different outcomes. Traditional prognostic models based on clinical and pathological parameters are often insufficient to fully capture this variability. In recent years, a wide range of biomarkers—derived from tumor tissue, blood samples, and molecular analyses—have been proposed to improve risk stratification. In this review, we bring together these diverse categories of biomarkers and discuss how they contribute to a more refined understanding of disease behavior. By highlighting both their potential and current limitations, we aim to provide a clearer framework for integrating these markers into clinical practice. Ultimately, a more accurate prognostic assessment could support better treatment decisions and more personalized patient care.

## 1. Introduction

As the predominant histological subtype of renal cell carcinoma, clear cell renal cell carcinoma (ccRCC) remains a major contributor to cancer-related morbidity and mortality worldwide [1,2]. Despite substantial advances in diagnostic imaging, surgical techniques, and systemic therapies, clinical outcomes are highly heterogeneous. This variability reflects pronounced intertumoral and intratumoral biological diversity that is incompletely explained by conventional clinicopathological models [3].

In patients with localized ccRCC, surgery with curative intent represents the standard of care; however, a clinically significant proportion will develop disease recurrence following nephrectomy [4]. Consequently, contemporary clinical practice relies on clinicopathological risk stratification systems—integrating parameters such as tumor stage, nucleolar grading, tumor size, and the presence of tumor necrosis—to estimate recurrence risk and guide postoperative surveillance strategies as well as adjuvant treatment considerations [5,6].

External validation studies continue to support the prognostic value of established models, including the Leibovich score, while simultaneously demonstrating persistent uncertainty, particularly among patients classified within intermediate-risk categories [7,8,9]. These limitations underscore the need for biomarkers that more precisely capture the biological determinants of tumor behavior beyond conventional morphologic assessment [10]. Building on these limitations, multimodal recurrence models that integrate heterogeneous data layers have demonstrated incremental predictive value beyond anatomic staging in localized disease [11,12,13]. Prognostic stratification is equally integral in metastatic ccRCC, where it informs clinical decision-making, treatment sequencing and trial design. The International Metastatic Renal Cell Carcinoma Database Consortium (IMDC) criteria remain the most widely adopted framework for categorizing patients into prognostic groups and guiding counseling and therapeutic strategies [14,15]. Nevertheless, substantial divergency in outcomes persists within IMDC-defined strata, indicating that key biological determinants of aggressive disease and treatment resistance are not fully encompassed by clinical variables alone [16].

In light of these challenges, constant efforts to improve prognostic assessment have increasingly turned toward the identification of biomarkers that can enhance risk stratification throughout all stages of the disease. Candidate approaches include tumor-intrinsic molecular alterations, such as genomic and transcriptomic features, tissue-based surrogate markers assessable by immunohistochemistry (IHC), characteristics of the tumor immune microenvironment, and circulating biomarkers that allow minimally invasive and longitudinal disease monitoring [6,16,17,18].

Importantly, the overarching objective is not limited to demonstrating statistically significant associations but rather to identify biomarkers—or integrated models—that deliver reproducible and clinically meaningful incremental prognostic value beyond existing tools, supported by standardized assays and prospective validation [19].

Starting from the current state of the art, this review summarizes and critically appraises the evidence published over the past decade regarding prognostic biomarkers in ccRCC. The analysis is structured into four overarching domains: (i) genomic biomarkers, covering somatic alterations and transcriptomic signatures; (ii) tissue-based biomarkers, including immunohistochemical surrogates and immune microenvironment features; (iii) circulating biomarkers, such as systemic inflammation parameters and indices; and (iv) integrated predictive models, represented by emerging multi-omic approaches.

Emphasis is placed on clinically relevant endpoints, including recurrence-free/disease-free survival (RFS/DFS), cancer-specific survival (CSS), overall survival (OS), and progression-free survival (PFS), as well as on the methodological and translational prerequisites required for implementation in routine clinical practice [6].

**Figure 1:**
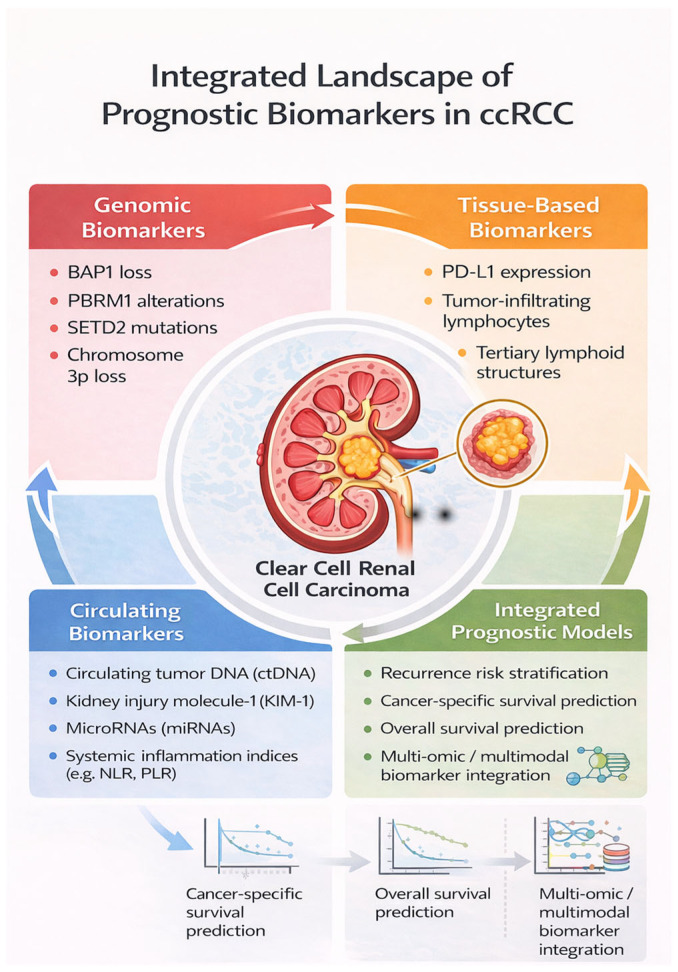
Integrated prognostic framework in ccRCC.

Figure 1 outlines the four principal domains of prognostic biomarkers in ccRCC: genomic alterations, tissue-based biomarkers, circulating biomarkers, and integrated prognostic models. These complementary layers collectively inform refined risk stratification for clinically relevant outcomes, including recurrence, cancer-specific survival, and overall survival.

## 2. Genomic Biomarkers

### 2.1. Genomic Prognostic Biomarkers

Genomic alterations constitute a central driver of biological heterogeneity in ccRCC. Large-scale sequencing efforts have demonstrated that, despite a relatively low overall mutational burden, ccRCC is characterized by recurrent and non-random alterations affecting genes involved in chromatin remodeling, epigenetic regulation and hypoxia signaling pathways [20,21,22]. These molecular features shape tumor behavior and have been extensively investigated for their prognostic relevance across disease stages [16,23].

#### 2.1.1. Chromosome 3p Alterations: *VHL, BAP1, PBRM1* and *SETD2*

Loss of chromosome 3p represents an early and near-universal event in ccRCC, resulting in the inactivation of multiple tumor suppressor genes located within this region, most notably Von Hippel–Lindau (*VHL*), Polybromo 1 (*PBRM1*), BRCA1-associated Protein 1 (*BAP1*) and SET domain containing 2 (*SETD2*) [21,22]. Among these, alterations involving *BAP1* have shown the most consistent association with aggressive tumor biology and adverse clinical outcomes.

Inactivation of VHL is identified in up to 90% of ccRCC and represents a central event in tumorigenesis through the activation of hypoxia-related and angiogenic pathways [23,24]. This molecular alteration contributes to the development of highly vascular tumors, a defining feature of ccRCC biology. The VHL gene encodes a component of a multiprotein complex responsible for targeting hypoxia-inducible factor (HIF) α subunits (HIF-1α, HIF-2α, and HIF-3α) for degradation under normoxic conditions [25]. This oxygen-dependent interaction between pVHL and HIFα is a key mechanism of cellular oxygen sensing. Under conditions of VHL dysfunction or reduced oxygen availability, HIFα escapes degradation, accumulates, and activates the transcription of genes involved in angiogenesis and metabolic adaptation [26,27,28,29]. Several independent retrospective cohort studies across localized and advanced ccRCC have consistently demonstrated that *BAP1* loss or mutation correlates with high nuclear grade, tumor necrosis, advanced pathological stage and reduced cancer-specific and overall survival [30,31,32,33,34]. Importantly, the adverse prognostic impact of *BAP1* inactivation has been observed even in clinically low-risk tumors and has remained significant in multi-variable analyses adjusted for established clinicopathological factors [31,33]. Collectively, these findings support *BAP1* as a robust genomic marker of aggressive disease biology in ccRCC.

In contrast, the prognostic significance of *PBRM1*, the most frequently mutated gene in ccRCC, appears more context-dependent. Several studies have suggested that *PBRM1*-mutated tumors may be associated with a more indolent clinical course, particularly in advanced disease, whereas others have failed to demonstrate independent prognostic value after adjustment for co-occurring genomic alterations [16,29,34]. This variability likely reflects complex interactions between *PBRM1* status and other genomic events, including its frequent mutual exclusivity with *BAP1* alterations [21,30,31].

Alterations in *SETD2*, a histone methyltransferase responsible for trimethylation of histone H3 at lysine 36 (H3K36me3), have been associated with increased genomic instability and a more aggressive tumor phenotype. Loss-of-function mutations in *SETD2* and the resulting epigenetic dysregulation have been linked to advanced pathological stages and adverse clinical outcomes in several cohorts [21,22].

#### 2.1.2. Non-3p Genomic Alterations and Pathway-Level Associations

Beyond chromosome 3p, additional genomic events have been explored for prognostic relevance in ccRCC. Alterations affecting components of the PI3K–AKT–mTOR axis, cell-cycle regulators and DNA damage response pathways occur in molecular subsets and may contribute to aggressive behavior in selected contexts [35,36]. However, the relatively low prevalence and context-dependent effects of many of these events limit their utility as standalone prognostic biomarkers in routine practice. Pathway-oriented approaches further emphasize that dysregulation of chromatin remodeling and epigenetic programs represents a recurrent biological theme in ccRCC progression, supporting a shift from single-gene markers toward integrated genomic and pathway-level profiles that better reflect disease complexity [35].

#### 2.1.3. Composite Mutational Profiles and Genomic Risk Models

Acknowledging the limitations inherent to individual genomic alterations, several studies have proposed composite mutational profiles that integrate multiple recurrently altered genes [21,37,38]. Panels incorporating *VHL*, *PBRM1*, *BAP1*, *SETD2*, *KDM5C*, mTOR and TP53 have demonstrated the ability to stratify patients into prognostically distinct categories, particularly in the setting of localized disease following nephrectomy [32].

Within these models, mutational patterns defined by *BAP1* inactivation in combination with additional alterations affecting chromatin remodeling pathways consistently delineate patient subsets characterized by an increased risk of disease relapse and inferior cancer-specific outcomes, even after adjustment for pathological stage and tumor grade [16,30].

In contrast, tumors harboring *PBRM1* alterations in the absence of *BAP1* inactivation tend to be associated with more favorable clinical outcomes, reinforcing the concept of biologically distinct molecular subtypes within ccRCC [34].

More broadly, evolutionary genomic studies further emphasize the complexity of these mutational interactions, demonstrating that tumor progression in ccRCC follows constrained evolutionary trajectories shaped by early truncal events and subsequent branching alterations [39].

#### 2.1.4. Localized Versus Metastatic Disease

The prognostic implications of genomic biomarkers differ substantially between localized and metastatic ccRCC. In localized disease, genomic alterations primarily inform the risk of postoperative recurrence and cancer-specific mortality, with *BAP1* loss emerging as the most reproducible adverse prognostic marker across studies [31,38]. In contrast, in the metastatic setting, genomic features appear to reflect broader aspects of tumor aggressiveness and may interact with treatment response; however, their independent prognostic value remains less clearly defined [36].

These observations emphasize that genomic biomarkers in ccRCC cannot be interpreted in isolation from disease stage, molecular background, and treatment exposure [16,21]. While recurrent genomic alterations have substantially refined the biological taxonomy of ccRCC, their prognostic impact is rarely autonomous and is frequently modulated by co-occurring mutations and evolving therapeutic landscapes [22,30]. Consequently, translation into clinical decision-making requires not only stage-specific validation but also integration within broader molecular and clinical frameworks [36].

The main genomic alterations with established or emerging prognostic relevance in ccRCC are summarized in Table 1.

### 2.2. Transcriptomic Signatures and Multigene Prognostic Models

Transcriptomic profiling has substantially advanced the understanding of biological heterogeneity in ccRCC, extending beyond single-gene alterations to capture coordinated patterns of gene expression associated with tumor behavior. Large-scale expression analyses have demonstrated that ccRCC can be subdivided into molecularly distinct subtypes with characteristic biological features and divergent clinical outcomes, supporting the prognostic potential of transcriptomic signatures [21,41].

#### 2.2.1. Molecular Subtypes Defined by Gene Expression

Early transcriptomic investigations identified ccRCC subgroups characterized by differential activation of pathways related to hypoxia signaling, angiogenesis, cellular metabolism and immune regulation [41,42]. Tumors enriched for cell-cycle progression and proliferative gene programs have been consistently associated with adverse clinical outcomes, whereas subtypes retaining features of metabolic differentiation generally exhibit more favorable prognoses [20,42].

Although these molecular subtypes show partial overlap with established clinico-pathological parameters, several analyses have demonstrated that transcriptomic classifications retain independent prognostic significance after multivariable adjustment. This observation suggests that gene expression–based stratification captures biological dimensions not fully reflected by tumor stage or grade alone [41].

#### 2.2.2. Prognostic Gene Expression Signatures

Building upon subtype classification, multiple studies have proposed multigene expression signatures aimed at predicting recurrence or survival outcomes in ccRCC. These signatures commonly incorporate genes involved in angiogenesis, immune response, extracellular matrix remodeling and epigenetic regulation. In localized disease, several expression-based risk scores (e.g., ClearCode34 and the 16-gene recurrence assay) stratify patients into prognostically distinct groups with differing recurrence-related and survival outcomes following nephrectomy [43,44].

More recent transcriptomic analyses have emphasized multigene prognostic signatures that outperform individual biomarkers. In localized ccRCC, a 17-gene expression signature was shown to discriminate patient subgroups with significantly different RFS/DFS and OS, suggesting improved molecular risk prediction compared with conventional staging alone [45]. Complementary studies have further expanded these approaches, demonstrating that integrated gene expression models can enhance prognostic accuracy beyond established clinical variables, thereby reinforcing the potential value of transcriptomic profiling for risk stratification in ccRCC [46].

Despite these encouraging findings, substantial heterogeneity exists among reported transcriptomic signatures, including variability in gene composition, analytical platforms and scoring methodologies. Consequently, direct comparisons across studies remain challenging, and only a limited number of signatures have undergone independent external validation to date [41].

#### 2.2.3. Integration with Genomic Alterations

Transcriptomic patterns in ccRCC are tightly coupled to underlying genomic alterations. Tumors harboring *BAP1* loss or mutation exhibit distinct gene expression profiles characterized by activation of inflammatory and proliferative pathways, which may partly account for their consistently adverse prognostic associations [18,47]. By contrast, *PBRM1*-altered tumors display transcriptional programs suggestive of altered chromatin accessibility and remodeling and, in specific biological and clinical contexts, may be associated with a more indolent disease course [29]. Collectively, these findings support an integrative molecular framework in which genomic and transcriptomic layers are interpreted synergistically to define biologically coherent and clinically relevant subgroups.

#### 2.2.4. Immune-Related Transcriptomic Signatures

Transcriptomic analyses in ccRCC have uncovered immune-related gene-expression signatures that carry important prognostic weight. In addition to tumor-intrinsic transcriptional programs, multiple studies have reported profiles enriched for interferon-α/γ signaling, inflammatory pathways, checkpoint-associated genes and markers of immune-cell infiltration [20,21]. In patients who have not received therapy, these “immune-inflamed” signatures are often linked to more aggressive disease and to shorter OS and PFS, suggesting that they reflect an exhausted or dysfunctional immune microenvironment rather than effective anti-tumor immunity [20,21,48].

Paradoxically, the same inflammatory transcriptional landscape can signal a tumor’s susceptibility to immune-checkpoint blockade, highlighting the dual and context-dependent nature of these signatures [20,21,48]. Consequently, it is essential to differentiate immune-related transcriptomic patterns that serve purely as prognostic markers from those that also predict therapeutic response. This distinction is particularly relevant in the contemporary therapeutic landscape of ccRCC, where immune checkpoint inhibitors have reshaped outcome expectations and may modify the prognostic value of baseline immune signatures [17].

Furthermore, most available data derive from retrospective or exploratory transcriptomic analyses, underscoring the need for prospective validation and standardized analytical frameworks before routine clinical implementation [20,31,47,48,49].

## 3. Tissue-Based Biomarkers

### 3.1. Tissue-Based Biomarkers and Immunohistochemical Surrogates

Tissue-based biomarkers continue to occupy a central role in prognostic research for ccRCC, owing to their direct assessment of tumor biology and their feasibility within routine diagnostic pathology workflows. IHC, in particular, has been extensively investigated as a practical surrogate for underlying genomic and epigenetic alterations, providing an important translational link between molecular discoveries and clinical implementation [29,31,33].

#### 3.1.1. *BAP1* Loss as an Immunohistochemical Prognostic Marker

Among tissue-based biomarkers in ccRCC, loss of nuclear *BAP1* expression has emerged as one of the most consistent and clinically relevant IHC findings. Across multiple retrospective cohorts, *BAP1* loss has been repeatedly associated with adverse clinicopathological features, including high International Society of Urological Pathology nucleolar grading, tumor necrosis, sarcomatoid dedifferentiation, and advanced pathological stage [31,33]. Notably, the prognostic impact of *BAP1* loss extends beyond tumors already classified as high-risk by conventional parameters. In patients with localized ccRCC, absence of *BAP1* expression has been linked to an increased risk of postoperative recurrence and reduced cancer-specific survival. Importantly, this association persists even after adjustment for established prognostic variables in multivariable models [31,32,50]. Taken together, these data position *BAP1* IHC not merely as a descriptive molecular correlate but as a practical surrogate of biologically aggressive disease. Its reproducibility, relative technical simplicity, and consistent prognostic signal support its potential integration into postoperative risk assessment frameworks.

#### 3.1.2. *PBRM1* Expression and Prognostic Heterogeneity

In contrast to *BAP1*, IHC assessment of *PBRM1* has yielded heterogeneous and context-dependent prognostic results in ccRCC. While loss of *PBRM1* expression frequently reflects underlying loss-of-function genomic alterations, its association with clinical outcomes has been inconsistent across studies. Several analyses have reported no independent prognostic significance for *PBRM1* expression after adjustment for established clinicopathological parameters [34]. Conversely, other studies have suggested that preserved or high *PBRM1* expression may be associated with more aggressive tumor behavior in selected clinical settings, further underscoring its context-specific biological role [29]. This variability likely reflects the complex biological functions of *PBRM1* in chromatin remodeling and transcriptional regulation, as well as its interaction with co-occurring genomic events, most notably *BAP1* status. Accordingly, *PBRM1* expression alone is unlikely to serve as a reliable standalone prognostic biomarker. However, when interpreted within a broader molecular or IHC context—particularly in combination with other chromatin remodeling alterations—*PBRM1* tissue-based assessment may provide incremental biological and prognostic information [29,34].

#### 3.1.3. *SETD2*/H3K36me3 Axis as an Epigenetic Surrogate

Alterations affecting the *SETD2*/H3K36me3 axis represent another epigenetic dimension of chromatin remodeling dysregulation in ccRCC. Loss of *SETD2* function leads to depletion of the H3K36me3 histone mark, which can be evaluated immunohistochemically and has been proposed as a surrogate of underlying genomic alterations [22]. Reduced or absent H3K36me3 expression has been associated with genomic instability, impaired DNA damage repair, and alterations in transcriptional regulation, reflecting the broader epigenetic consequences of *SETD2* inactivation. Several studies have suggested that disruption of the *SETD2*/*H3K36me3* pathway may correlate with more advanced pathological stages and unfavorable clinical outcomes, although the strength and independence of this association appear less consistent than those observed for *BAP1* loss [22,40].

Importantly, *H3K36me3* IHC offers a practical approach to approximate *SETD2* functional status in routine pathology practice. Compared with genomic sequencing, assessment of the *H3K36me3* mark provides a readily applicable method to detect downstream epigenetic consequences of *SETD2* alterations directly within tumor tissue. Nevertheless, variability in staining patterns and the complex interplay between *SETD2* and other chromatin remodeling genes suggest that this marker may be most informative when interpreted within integrated molecular or immunohistochemical panels rather than as an isolated prognostic indicator [22,40].

#### 3.1.4. Integrated Immunohistochemical Panels

Recognizing the limitations inherent to single-marker strategies, more recent work has explored integrated immunohistochemical panels combining *BAP1*, *PBRM1*, and H3K36me3 expression in an effort to better approximate the underlying molecular architecture of ccRCC [31,32,40]. Rather than evaluating these markers in isolation, panel-based approaches aim to capture coordinated patterns of chromatin remodeling alterations that define biologically distinct subgroups. Within this framework, tumors characterized by *BAP1* loss—particularly in the setting of preserved *PBRM1* expression—have been consistently linked to more aggressive pathological features and poorer clinical outcomes [32,33,40]. By contrast, alternative expression constellations within these panels tend to align with comparatively indolent clinical trajectories [32,33]

These findings suggest that combinatorial IHC assessment may offer greater biological resolution than single-marker evaluation. Importantly, such panels provide a practical bridge between morphology and genomics: they approximate complex molecular landscapes using techniques that are widely available in routine pathology laboratories. In settings where comprehensive molecular profiling is not feasible, integrated IHC panels therefore represent a pragmatic and scalable strategy to refine prognostic stratification.

Table 2 summarizes key tissue-based prognostic biomarkers in ccRCC, their molecular surrogates, IHC evaluation strategies, and associated prognostic significance.

### 3.2. Immune Microenvironment–Related Prognostic Biomarkers

ccRCC is characterized by a highly complex and dynamic tumor immune microenvironment that plays a critical role in tumor progression and clinical outcomes. Beyond its established relevance for predicting response to immunotherapy, accumulating evidence indicates that immune-related features of the tumor microenvironment also convey intrinsic prognostic information, independent of treatment exposure [20,51,52,53,54,55].

#### 3.2.1. PD-L1 Expression

Programmed death-ligand 1 (PD-L1) expression has been extensively investigated in ccRCC as both a prognostic and biologically informative biomarker. Across multiple studies, PD-L1 positivity—whether assessed on tumor cells or tumor-infiltrating immune cells—has been associated with adverse clinicopathological features, including higher tumor grade, advanced pathological stage, and reduced overall and cancer-specific survival [52,53].

In localized disease, PD-L1 expression has been linked to an increased risk of postoperative relapse in several retrospective cohorts [52,53,54]. However, its prognostic contribution appears to overlap substantially with established clinicopathological parameters, and its independent effect is often attenuated in multivariable models [20,52]. This suggests that PD-L1 may function less as an autonomous prognostic driver and more as a biological correlate of aggressive tumor phenotypes.

In metastatic ccRCC, PD-L1 positivity generally reflects an inflamed and high-risk tumor microenvironment characterized by increased immune cell infiltration and activation of inhibitory immune pathways [51,52]. In the absence of systemic therapy, this expression pattern has been associated with inferior outcomes, reinforcing its link to aggressive disease biology [53,54]. Thus, while PD-L1 expression consistently aligns with adverse clinical features, its role as a standalone prognostic marker remains intertwined with the broader tumor and immune context. Importantly, this context-dependent role may also explain why, in contrast to other malignancies, immune checkpoint inhibitors in ccRCC are currently administered irrespective of PD-L1 expression status, as clinical benefit has been observed across PD-L1 subgroups [51,52,53,54,55].

#### 3.2.2. Tumor-Infiltrating Lymphocytes and Immune Exhaustion

The prognostic significance of tumor-infiltrating lymphocytes (TILs) in ccRCC differs substantially from the paradigm observed in many other solid tumors. In contrast to malignancies where abundant CD8-positive T-cell infiltration often signals effective antitumor immunity, ccRCC demonstrates a more complex and sometimes counterintuitive pattern. Several studies have shown that a high density of CD8-positive T cells is not reliably associated with improved outcomes; instead, increased T-cell infiltration frequently correlates with markers of immune dysfunction and exhaustion, including upregulation of inhibitory immune checkpoint pathways, and has been linked to adverse prognosis [51].

This paradox likely reflects the presence of a chronically stimulated yet functionally impaired immune response. Persistent antigen exposure, sustained interferon signaling, and progressive loss of cytotoxic effector capacity contribute to a state in which T cells are present but biologically ineffective [20,51]. In this setting, lymphocyte density alone does not equate to immune competence.

Accordingly, quantitative assessment of TILs appears insufficient as an isolated prognostic metric in ccRCC. Greater insight may be gained from functional characterization of immune states—distinguishing active, inflamed microenvironments from exhausted or immunosuppressed phenotypes—rather than relying solely on numerical counts [20].

#### 3.2.3. Tertiary Lymphoid Structures

Tertiary lymphoid structures (TLS) are organized aggregates of immune cells that recapitulate features of secondary lymphoid organs and may facilitate local antitumor immune responses. In ccRCC, emerging evidence indicates that prognostic relevance depends not merely on the presence of TLS but on their spatial organization and degree of maturation [56].

Well-structured, mature TLS—characterized by distinct B-cell zones, T-cell areas, and follicular dendritic cell networks—has been associated with improved survival outcomes. In contrast, diffuse or poorly organized lymphoid aggregates tend to correlate with less favorable clinical trajectories [56]. These findings underscore that immune architecture, rather than immune cell abundance alone, shapes the biological impact of the tumor microenvironment.

#### 3.2.4. Functional Immune Signaling and Emerging Pathways (STING)

Beyond established cellular and structural components of the tumor immune microenvironment, emerging evidence highlights the role of functional immune signaling pathways in shaping tumor behavior and clinical outcomes in ccRCC.

Recent studies have also implicated alterations in the cGAS–STING pathway in influencing prognosis, shaping immune signatures—particularly through IL6-related signaling—and modulating therapeutic responses in ccRCC and other renal cell neoplasms [57,58]. Emerging evidence suggests that STING-related signaling may not necessarily reflect effective antitumor immunity, but rather a qualitatively altered inflammatory state associated with tumor progression [59]. This concept is supported by observations in other renal tumor subtypes, such as renal medullary carcinoma, which exhibits a highly inflamed yet immunosuppressive microenvironment characterized by abundant immune infiltration and upregulation of multiple immune checkpoints in the context of cGAS–STING pathway activation [60].

Similarly, in fumarate hydratase (FH)–deficient renal cell carcinoma, STING expression has been associated with aggressive clinical behavior and frequently coexists with PD-L1–positive, inflamed tumor phenotypes, suggesting both prognostic and potential predictive relevance [61].

Taken together, these findings highlight the cGAS–STING axis as a potential link between tumorigenesis, immune infiltrate, and tumor biology and suggest that immune-related biomarkers in ccRCC should be interpreted within a broader functional and signaling context.

#### 3.2.5. Interpretation and Clinical Relevance 

Collectively, biomarkers related to the immune microenvironment add an important spatial and functional dimension to prognostic assessment in ccRCC. Their interpretation, however, requires careful consideration of morphological context, structural organization, and intratumoral heterogeneity.

Importantly, the prognostic value of these biomarkers does not rely solely on their presence or abundance, but rather on the functional state of the immune microenvironment, including patterns of immune activation, suppression, and spatial organization.

Key immune-related tissue biomarkers with reported prognostic relevance, together with their principal interpretative considerations, are summarized in Table 3.

## 4. Circulating Biomarkers

### 4.1. Specific Circulating Prognostic Biomarkers

Circulating biomarkers have attracted growing interest in ccRCC because of their minimally invasive nature and their capacity to capture dynamic aspects of tumor biology over time. Unlike tissue-based markers obtained at a single time point, circulating molecules may reflect systemic tumor burden, evolving biological behavior, and residual disease activity. As such, they offer prognostic information that complements conventional pathological assessment across different stages of disease [20].

#### 4.1.1. Kidney Injury Molecule-1

Kidney injury molecule-1 (KIM-1) is among the most extensively validated circulating biomarkers with demonstrated prognostic relevance in ccRCC. Although initially described as a marker of renal tubular injury, KIM-1 is markedly overexpressed in ccRCC tumor cells and can be detected in both serum and urine, making it particularly attractive for longitudinal monitoring [62,63].

Clinical studies have consistently shown that elevated circulating KIM-1 levels correlate with advanced pathological stage, increased tumor burden, and adverse clinical outcomes. In patients with localized ccRCC, higher plasma KIM-1 concentrations have been independently associated with an increased risk of recurrence and reduced cancer-specific survival following nephrectomy, even after adjustment for established clinicopathological risk models [63]. These findings suggest that circulating KIM-1 may capture biological aggressiveness or microscopic residual disease not fully accounted for by conventional staging systems.

In metastatic disease, elevated circulating KIM-1 levels have similarly been associated with inferior overall survival, supporting their role as markers of systemic tumor burden [63]. Interestingly, transcriptomic analyses indicate that higher tumoral *HAVCR1* (KIM-1) mRNA expression may correlate with improved overall survival in certain contexts. Mechanistic studies have provided a potential explanation for this apparent paradox: experimental work has demonstrated that KIM-1 can downregulate the pro-metastatic gene Rab27b, thereby reducing pulmonary metastases in preclinical models [64,65]. Together, these observations highlight the complex and context-dependent biological functions of KIM-1 in ccRCC progression.

#### 4.1.2. Circulating microRNAs

Circulating microRNAs (miRNAs) have emerged as candidate prognostic biomarkers owing to their remarkable stability in peripheral blood and their central role in post-transcriptional gene regulation [66,67,68,69]. Their resistance to degradation and detectability in plasma or serum have supported their evaluation as minimally invasive biomarkers across multiple malignancies, including ccRCC.

In ccRCC, several circulating miRNAs have been linked to aggressive tumor phenotypes and unfavorable clinical outcomes [7,69]. Distinct expression patterns have been associated with higher tumor grade, advanced pathological stage, and reduced overall and cancer-specific survival [47,68,69]. Importantly, some of these miRNA profiles appear to mirror intrinsic tumor biology rather than representing nonspecific inflammatory or systemic responses.

Integrative analyses have further demonstrated associations between specific circulating miRNAs and recurrent genomic alterations in ccRCC, including *BAP1* inactivation [16,47]. These correlations suggest that certain circulating miRNA signatures may function as peripheral readouts of underlying molecular subtypes characterized by adverse prognosis [47].

At the same time, interpretation of the current literature requires caution. Reported signatures vary considerably across studies, reflecting differences in patient selection, sample processing, normalization strategies, and analytical platforms. Consequently, inter-study concordance remains modest, and no single circulating miRNA panel has consistently reproduced prognostic performance across independent cohorts [20,69]. In this context, circulating miRNAs currently represent a biologically compelling but analytically heterogeneous class of biomarkers, whose translational trajectory will depend on methodological harmonization and rigorous validation.

#### 4.1.3. Circulating Tumor DNA

The analysis of circulating tumor DNA (ctDNA) has emerged as a technologically promising approach for prognostic assessment, minimal residual disease (MRD) detection, and dynamic disease monitoring in ccRCC. However, in contrast to malignancies characterized by high levels of tumor DNA shedding, ccRCC typically releases relatively low quantities of tumor-derived DNA into the circulation. This biological feature inherently limits analytical sensitivity, particularly in localized and early-stage disease, where detection rates remain modest [69,70,71].

Despite these constraints, available data indicate that detectable ctDNA carries meaningful prognostic information. The presence of ctDNA has been associated with advanced pathological stage and adverse clinical outcomes [36,70]. In the postoperative setting, ctDNA positivity following curative-intent surgery identifies patients at substantially increased risk of recurrence and reduced survival, underscoring its potential relevance for dynamic risk stratification and surveillance [70].

Clinical integration of ctDNA in ccRCC remains influenced by both biological and technical variables. Reported performance varies according to sequencing depth, assay design, and bioinformatic pipelines, and consensus regarding optimal sampling timing and clinically actionable thresholds has yet to be established [36,70]. Collectively, these considerations position ctDNA as a biologically informative yet stage-sensitive biomarker, whose greatest utility may reside in identifying high-risk patients and monitoring disease evolution rather than in broad population-level screening.

#### 4.1.4. Other Circulating Biomarkers

Several circulating molecules have been explored as potential prognostic biomarkers in ccRCC. Among soluble immune-regulatory proteins, elevated circulating soluble PD-L1 and soluble PD-1 levels have been associated with adverse clinicopathological features and inferior survival outcomes in selected cohorts of metastatic disease [72,73].

Circulating interleukin-6 (IL-6), a key mediator of systemic inflammation, has likewise been linked to unfavorable outcomes in advanced RCC. In patients treated with immune checkpoint inhibitor–based combinations, elevated baseline IL-6 levels correlated with reduced overall and progression-free survival [74]. Similarly, in metastatic ccRCC treated with targeted therapy, higher plasma IL-6 levels were associated with shorter progression-free survival [75].

Pro-angiogenic factors such as vascular endothelial growth factor A (VEGF-A) have also been investigated, with higher baseline circulating levels reflecting aggressive disease biology and inferior clinical outcomes in advanced RCC [76].

Hypoxia-related mediators, including carbonic anhydrase IX (CAIX), have been evaluated in association with prognosis, consistent with the central role of the Von Hippel–Lindau (VHL)–hypoxia-inducible factor (HIF) pathway dysregulation in ccRCC tumorigenesis [3,77].

Although these circulating markers provide biologically meaningful insights into tumor-associated inflammation, angiogenesis, and immune regulation, heterogeneity in assay platforms, cutoff definitions, and patient populations continues to limit cross-study comparability and standardization. As such, their integration into routine clinical prognostic models remains investigational.

#### 4.1.5. Clinical Relevance and Limitations

Despite these exploratory signals, none of these circulating biomarkers—whether inflammatory indices, soluble immune mediators, angiogenic factors, or ctDNA—have consistently demonstrated independent prognostic value after robust multivariable adjustment, nor have they undergone sufficient external validation to justify adoption as standalone prognostic tools in routine clinical practice [20,60]. Consequently, circulating biomarkers currently appear more suitable for dynamic disease monitoring and advanced-stage risk stratification rather than independent prognostic decision-making in localized ccRCC. The principal circulating biomarkers with reported prognostic relevance in ccRCC, together with their key methodological and interpretative considerations, are summarized in Table 4.

### 4.2. Systemic Inflammatory Biomarkers and Composite Prognostic Scores

Systemic inflammation is increasingly recognized as a key contributor to tumor progression and adverse clinical outcomes across solid malignancies, including ccRCC. Biomarkers derived from routine hematological parameters provide indirect insight into tumor–host interactions and have been widely investigated for prognostic relevance, largely due to their broad availability, low cost and ease of longitudinal assessment [78]. An overview of systemic biomarkers associated with prognostic outcomes in ccRCC, along with the main methodological and interpretative considerations affecting their evaluation, is presented in Table 4.

#### 4.2.1. Neutrophil-to-Lymphocyte Ratio

The neutrophil-to-lymphocyte ratio (NLR) is the most extensively investigated systemic inflammatory biomarker in ccRCC. Elevated NLR has been consistently associated with adverse clinical outcomes, including reduced OS and CSS, across both localized and metastatic disease settings [78].

In localized ccRCC, higher preoperative NLR values have been linked to an increased likelihood of postoperative recurrence and less favorable long-term outcomes. Notably, this association has persisted after adjustment for established clinicopathological prognostic factors in multivariable models. In metastatic disease, NLR has retained prognostic relevance across different therapeutic contexts, suggesting that it captures aspects of systemic inflammation and tumor–host interaction that extend beyond stage alone [20,78]. From a biological perspective, elevated NLR may reflect a relative expansion of pro-tumorigenic neutrophil populations together with reduced lymphocyte-mediated antitumor immunity, thereby functioning as a peripheral surrogate of systemic immune imbalance [20,78]. At the same time, reported cutoff thresholds, timing of measurement, and analytical strategies vary substantially across studies, limiting inter-study comparability and complicating standardization [20]. Within this context, NLR can be viewed as a readily accessible marker of systemic inflammatory tone whose prognostic value is biologically plausible but methodologically context-dependent.

#### 4.2.2. Platelet-to-Lymphocyte and Lymphocyte-to-Monocyte Ratios

Beyond NLR, additional systemic inflammatory indices have been explored in ccRCC, including the platelet-to-lymphocyte ratio (PLR) and the lymphocyte-to-monocyte ratio (LMR). Across multiple retrospective cohorts, elevated PLR and reduced LMR have been associated with adverse clinical outcomes, although their prognostic performance appears generally more modest and less consistent than that reported for NLR [20,78]. Biologically, thrombocytosis may reflect tumor-driven inflammatory signaling and platelet-mediated facilitation of tumor progression, including protection of circulating tumor cells and promotion of angiogenesis. Conversely, reduced lymphocyte counts may indicate impaired antitumor immune surveillance, while increased monocyte fractions can signal enhanced recruitment of pro-tumorigenic macrophages within the tumor microenvironment [79,80]. Nonetheless, these composite ratios capture only partial aspects of systemic immune dynamics, which may account for their variable and context-dependent prognostic strength in ccRCC.

#### 4.2.3. Systemic Immune-Inflammation Index

Building on single-ratio inflammatory markers, composite indices such as the systemic immune-inflammation index (SII)—calculated by integrating neutrophil, platelet, and lymphocyte counts—have been proposed as a more comprehensive representation of systemic inflammatory status [81,82]. By incorporating multiple cellular compartments, SII aims to better capture the dynamic interplay between protumor inflammatory activity and adaptive immune surveillance. Available data in RCC suggest that SII may provide incremental prognostic stratification compared with single-ratio markers such as NLR or PLR, particularly in advanced disease settings [82,83]. However, its comparative value appears to vary according to patient population, treatment context, and disease stage, underscoring the need for contextual interpretation.

#### 4.2.4. C-Reactive Protein and Inflammation-Based Scores

C-reactive protein (CRP), a widely available acute-phase reactant, has been extensively examined as a surrogate marker of systemic inflammation in ccRCC. Elevated CRP levels have been associated with advanced disease stage, adverse pathological features, and inferior survival outcomes in retrospective clinical analyses [20,78].

Composite inflammation-based indices incorporating CRP, such as the Glasgow Prognostic Score (GPS), have further reinforced the association between systemic inflammatory activation and oncologic prognosis in RCC [84,85]. By integrating CRP with additional laboratory parameters, these scores aim to contextualize inflammatory burden within broader host-response dynamics. However, CRP reflects generalized inflammatory activity rather than tumor-specific biological processes. Its levels may be influenced by comorbid conditions, infections, or other intercurrent inflammatory states, which complicates interpretation in individual patients [79]. In this sense, CRP-based scores provide a global measure of systemic inflammatory tone rather than a direct surrogate of tumor biology [78].

## 5. Integrated Prognostic Models

The modest prognostic performance of individual biomarkers in ccRCC has driven a conceptual shift toward integrated prognostic frameworks that combine multiple layers of biological and clinical information. Multi-omic approaches seek to more comprehensively capture tumor complexity by integrating genomic, transcriptomic, immune-related, circulating and imaging-derived features into conceptual unified risk models, thereby addressing the intrinsic heterogeneity of ccRCC [20,41].

### 5.1. Multi-Omic Prognostic Models

In recent years, prognostic modeling in ccRCC has evolved from single-gene or single-platform analyses toward integrative multi-omic frameworks. By combining recurrent genomic alterations—such as *BAP1*, *PBRM1*, and *SETD2*—with transcriptomic signatures and immune-related features, these models seek to more comprehensively represent the biological heterogeneity of the disease [16,21,37]. Analyses derived from large-scale public datasets, including The Cancer Genome Atlas, have delineated molecular subgroups defined by integrated genomic and gene expression profiles that display distinct survival patterns and clinical trajectories [21,38]. In localized ccRCC, integrated multi-omic models have been particularly explored in the postoperative setting. Their main contribution appears to lie in refining recurrence risk assessment among patients classified as intermediate risk by conventional clinicopathological systems. In this group, where tumor stage and grade alone often provide limited separation between favorable and unfavorable outcomes, molecular integration can help distinguish tumors with similar morphology but different biological potential [41].

Rather than replacing established clinicopathological predictors, multi-omic models seem to enrich them. Tumor stage primarily reflects the anatomical extent of disease, whereas genomic and transcriptional alterations provide insight into intrinsic biological behavior and evolutionary potential. When interpreted together, these complementary dimensions allow for a more refined biological stratification of ccRCC [21,38,41]. In this sense, multi-omic prognostic models represent an effort to integrate morphological assessment with molecular context, aligning traditional risk categories with underlying tumor biology.

### 5.2. Integration of Immune and Circulating Biomarkers

Recent multi-omic approaches in ccRCC have increasingly incorporated features of the tumor immune microenvironment alongside circulating biomarkers, reflecting the dynamic interaction between tumor genomics and host systemic responses. The inclusion of immune-related gene expression signatures, PD-L1 expression status, and systemic inflammatory indices into molecular risk models has demonstrated incremental improvements in prognostic discrimination in retrospective cohorts [20]. Rather than replacing genomic classifiers, these immune parameters appear to refine risk stratification by capturing variability in immune activation and exhaustion states that are not fully explained by tumor-intrinsic alterations.

Circulating biomarkers introduce a temporal dimension to prognostic modeling. KIM-1 has been associated with tumor burden and recurrence risk, while ctDNA provides insight into minimal residual disease and clonal dynamics following surgical resection [63,69]. In this context, circulating markers may complement baseline tissue-based profiling by reflecting ongoing biological activity rather than static molecular snapshots. At the same time, the added prognostic value of integrated models appears to be context-dependent. The magnitude of improvement over established clinicopathological scores is often modest, and model performance may vary across disease stages and therapeutic settings. This suggests that immune and circulating biomarkers are more likely to function as modulators of existing risk frameworks rather than as independent determinants of prognosis. Their optimal role may therefore lie in dynamic surveillance and therapy monitoring, particularly in high-risk or advanced-stage disease, where temporal biological changes are most clinically relevant [20,63,69].

### 5.3. Toward the Clinical Implementation of Prognostic Biomarkers in Clear Cell Renal Cell Carcinoma

Table 5 summarizes a comparative assessment of representative candidate prognostic biomarkers in ccRCC, structured according to key parameters relevant for clinical implementation. These include strength of evidence, reproducibility, assay standardization, incremental value beyond established clinicopathological models, and overall clinical maturity.

Among tissue-based biomarkers, BAP1 loss [30,31,33] emerges as one of the most robust candidates, supported by consistent evidence, high reproducibility, and relatively advanced clinical maturity. In contrast, *PBRM1* mutations [16,33] demonstrate moderate-to-high evidence but are limited by variability across cohorts and lower levels of assay standardization.

At the transcriptomic level, ClearCode34 signatures [37,41,42,43] provide strong prognostic stratification and high incremental value; however, their broader clinical adoption remains constrained by platform dependency and limited standardization. Immune-related tissue biomarkers such as PD-L1 expression [54] and TILs [51] show variable prognostic relevance, largely due to heterogeneity in assessment methods, including differences in scoring systems and cut-off definitions, as well as limited reproducibility across studies.

Circulating biomarkers offer a minimally invasive alternative, with KIM-1 [62,63,64] increasingly supported by consistent evidence and showing moderate clinical maturity. In contrast, ctDNA [70,71] represents an emerging approach with high potential incremental value, particularly for dynamic disease monitoring, albeit currently limited by technical and standardization challenges. In parallel, systemic inflammatory indices such as NLR and SII [78] are supported by consistent and relatively reproducible evidence, although their incremental prognostic value remains moderate.

Finally, integrated models [20,63,69], combining genomic, tissue-based, and circulating data, demonstrate high overall potential, particularly in terms of incremental prognostic value, reflecting the ongoing shift toward multi-dimensional and integrative prognostic frameworks, despite persistent challenges related to standardization and clinical implementation.

Overall, this comparative framework highlights the complementary nature of different biomarker categories and underscores the need for further prospective validation and methodological harmonization, particularly within biomarker-driven clinical studies, before routine clinical integration.

## 6. Limitations and Future Perspectives

Although the body of literature on prognostic biomarkers in ccRCC has expanded considerably, the available evidence remains markedly heterogeneous in terms of study design, patient populations, disease stage, therapeutic context, and clinical endpoints. Variability in assay methodologies, biomarker cut-offs, and analytical approaches further complicates cross-study comparisons and limits reproducibility [18,20]. In addition, most published data derive from retrospective cohorts, often lacking independent external validation, which restricts their clinical robustness.

Consequently, despite these advances, the routine clinical implementation of biomarkers in ccRCC remains limited. A central challenge lies in the pronounced biological heterogeneity of the disease, both between patients and within individual tumors. Intratumoral heterogeneity may introduce sampling bias—particularly for tissue-based biomarkers—and likely contributes to inconsistent findings across studies [29,86]. The predominance of retrospective study designs, involving heterogeneous patient populations, variable treatment exposures, and inconsistent endpoint definitions, further increases the risk of confounding and limits the generalizability of reported associations [20,41].

Methodological variability also complicates interpretation. Differences in assay platforms, antibody clones, scoring systems, and cut-off definitions affect immunohistochemical biomarkers, while transcriptomic and circulating analyses are similarly constrained by the lack of harmonized analytical frameworks [6]. In parallel, the incremental prognostic value of many proposed biomarkers over established clinicopathological models remains insufficiently demonstrated, as statistical significance does not necessarily translate into clinically meaningful improvements in risk stratification or decision-making [17].

Future research should prioritize prospective, multi-institutional validation studies incorporating standardized methodologies and clinically relevant endpoints, particularly in localized ccRCC, where improved risk stratification could inform adjuvant therapy selection and surveillance strategies [6,17]. The development of integrated prognostic models combining genomic, tissue-based, immune, and circulating biomarkers with established clinical parameters represents a promising direction. However, simplification of model architecture, assay harmonization, and demonstration of reproducibility and cost-effectiveness will be essential to ensure feasibility in routine practice.

Ultimately, the clinical adoption of novel biomarkers will depend not only on statistical associations but also on clear evidence that they meaningfully improve patient stratification, therapeutic decision-making, and outcome prediction in well-designed prospective studies [20].

## 7. Final Remarks

ccRCC is a biologically heterogeneous malignancy in which conventional clinicopathological models only partially explain outcome variability. Over the past decade, substantial advances have broadened the spectrum of candidate prognostic biomarkers across genomic, transcriptomic, tissue-based, immune, and circulating domains [20,29].

Among these, chromosome 3p–related genomic alterations—particularly *BAP1* loss—remain the most consistently validated indicators of aggressive tumor biology. Immunohistochemical assessment of *BAP1* expression offers a pragmatic and reproducible surrogate for underlying genomic events and currently represents one of the most clinically mature molecular biomarkers in ccRCC [31,33]. Other molecular and immune-related markers, including *PBRM1*, the *SETD2*/H3K36me3 axis, and transcriptomic immune signatures, provide important biological insights but exhibit variable and context-dependent prognostic performance when evaluated in isolation.

Circulating biomarkers and systemic inflammatory indices provide minimally invasive options for longitudinal assessment, particularly after nephrectomy [63,78]. Yet, because they primarily capture systemic inflammatory tone or overall disease burden rather than tumor-intrinsic biology, their prognostic specificity remains limited. Consequently, they should be regarded as supportive indicators rather than independent determinants of clinical risk.

Looking forward, meaningful progress in prognostic stratification will likely depend on integrative models that combine genomic alterations, tissue-based biomarkers, immune context, and circulating parameters with established clinical variables. Such multimodal frameworks hold promise for more refined and biologically informed risk assessment. Nevertheless, until prospective validation and clear demonstration of incremental clinical utility are achieved, novel biomarkers should be considered enhancers of biological stratification rather than standalone arbiters of therapeutic decision-making [6].

Ultimately, improving prognostic precision in ccRCC will require not only continued biomarker discovery, but also rigorous validation, methodological harmonization, and careful integration into clinically actionable frameworks that demonstrably improve patient outcomes.

## 8. Conclusions

Prognostic stratification in clear cell renal cell carcinoma is undergoing a significant transformation, driven by the expanding landscape of genomic, tissue-based, and circulating biomarkers. While each category provides valuable insights into tumor biology and clinical behavior, none is sufficient as a standalone predictor. Instead, their greatest potential lies in integrative approaches that combine molecular alterations, tumor microenvironment features, and systemic biomarkers with established clinicopathological parameters.

Despite promising advances, several challenges remain, including limited standardization, variable reproducibility, and the need for robust prospective validation. Future efforts should focus on the development of clinically applicable, multi-dimensional models capable of guiding personalized management. Such approaches may ultimately bridge the gap between biological complexity and practical decision-making, improving patient outcomes in this heterogeneous disease.

## Figures and Tables

**Table 1:** Genomic prognostic biomarkers and mutational profiles in clear cell renal cell carcinoma.

Biomarker/Genomic Alteration (Key References)	Chromosomal Location/Pathway	Biological Function	Prognostic Significance
Chromosome 3p loss [21,22]	3p	Early truncal genomic events leading to inactivation of tumor suppressor genes (*VHL*, *PBRM1*, *BAP1*, *SETD2*)	Foundational genomic event underlying ccRCC tumorigenesis and biological heterogeneity
*BAP1* [31]	3p21.1	Chromatin remodeling and DNA damage response	Strongly associated with high nucleolar grading, tumor necrosis, advanced pathological stage and reduced cancer-specific and overall survival
*PBRM1* [16,29,34]	3p21.1	SWI/SNF chromatin remodeling complex	Prognostic impact is context-dependent; it is frequently associated with more indolent disease in the absence of *BAP1* alterations
*SETD2* [22,40]	3p21.31	Histone H3K36 trimethylation and epigenetic regulation	Associated with genomic instability and advanced disease; prognostic value less consistent than *BAP1*
Non-3p genomic alterations [35,36]	PI3K–AKT–mTOR, cell cycle, DDR	Regulation of cell growth and survival	Context-dependent prognostic impact; limited value as standalone biomarkers
Epigenetic/chromatin remodeling pathways [35]	Chromatin regulatory programs	Transcriptional and epigenetic regulation	Recurrent dysregulation supporting pathway-level prognostic assessment
Composite mutational profiles [38]	Multi-gene panels	Integrated genomic risk stratification	Stratify patients into prognostic categories after nephrectomy
*BAP1*-driven genomic profile [38]	Chromatin remodeling pathways	Aggressive molecular subtype	Increased relapse risk and poorer cancer-specific survival
*PBRM1*-mutated/*BAP1*-wild-type profile [34]	Chromatin remodeling pathways	Less aggressive molecular subtype	Generally associated with more favorable clinical outcomes
Stage-specific genomic impact [31,36]	Localized vs metastatic disease	Context-dependent biological behavior	Stronger prognostic value in localized disease

**Table 2:** Tissue-based immunohistochemical prognostic biomarkers in ccRCC.

Biomarker (Key References)	Molecular Alteration	IHC Evaluation	Prognostic Significance
*BAP1* [31,32,50]	Chromosome 3p tumor suppressor loss	Loss of nuclear *BAP1* staining	Strongly associated with high grade, tumor necrosis, advanced stage, postoperative relapse, and reduced cancer-specific survival; independent prognostic value
*PBRM1* [29,34]	Chromatin remodeling alterations	Loss vs retained nuclear expression	Context-dependent prognostic impact; limited value as a standalone marker; informative when interpreted in combination with other biomarkers
H3K36me3 [40]	*SETD2* inactivation	Loss or reduction in nuclear staining	Associated with adverse pathological features and poorer outcomes; modest and inconsistent independent prognostic value
Integrated IHC panels [31,32,33]	Combined chromatin and epigenetic dysregulation	Multiparametric interpretation	Stratify tumors into aggressive versus indolent biological subgroups; *BAP1* loss–driven profiles are associated with unfavorable prognosis

**Table 3:** Immune microenvironment–related prognostic biomarkers in ccRCC.

Biomarker (Key References)	Assessment	Prognostic Impact	Key Caveat
PD-L1 [52,53]	IHC (tumor and/or immune cells)	Associated with adverse outcomes and reduced overall and cancer-specific survival	Effect often not independent; assay-dependent
CD8^+^ TILs [51]	IHC density	High density is often associated with immune exhaustion and adverse outcomes	Functional state is more important than quantity
Tertiary lymphoid structures (TLS) [56]	Histology/IHC	Mature TLS associated with improved survival outcomes	Prognostic impact depends on maturation and spatial organization
Immune transcriptomic signatures [20,21]	Transcriptomics	Immune-inflamed profiles linked to aggressive disease biology	Prognostic significance does not necessarily predict therapeutic response

**Table 4:** Circulating and systemic prognostic biomarkers in ccRCC.

Biomarker (Key References)	Assessment	Prognostic Impact	Key Caveat
KIM-1 [62,63]	Serum/urine	Associated with advanced stage, increased recurrence risk and reduced survival in localized and metastatic ccRCC	Assay heterogeneity; no standardized cut-offs
Circulating miRNAs [20,47,69]	Plasma/serum	Associated with aggressive tumor phenotypes and poor survival; selected signatures linked to *BAP1* loss	Small cohorts; limited reproducibility
ctDNA [70,71]	Plasma	Detectable ctDNA associated with higher recurrence risk and inferior survival in selected cohorts	Low detection rates in localized disease; investigational
NLR [78]	Peripheral blood	Elevated values associated with poor survival and increased recurrence risk	Variable cut-offs; timing dependent
PLR, LMR and SII [20,78]	Peripheral blood	Associated with adverse outcomes; SII may improve prognostic stratification	Limited validation; heterogeneous methodologies
CRP [78]	Serum	Elevated levels correlate with poor prognosis	Low tumor specificity; influenced by comorbid inflammation

**Table 5:** Comparative assessment of candidate prognostic biomarkers in clear cell renal cell carcinoma according to key parameters relevant for clinical implementation.

Biomarker/ Model	Category	Strength of Evidence	Reproducibility	Assay Standardization	Incremental Value	Clinical Maturity
BAP1 loss [30,31,33]	Tissue-based (IHC)/Genomic	High	High	Moderate–High	Moderate–High	High
*PBRM1* mutation [16,33]	Genomic	Moderate–High	Moderate	Low–Moderate	Moderate	Moderate
Transcriptomic signatures—ClearCode34 [37,41,42,43]	Genomic/Integrated	High	Moderate	Low	High	Moderate
PD-L1 expression [54]	Tissue-based (IHC)	Moderate	Low–Moderate	Low	Variable	Low–Moderate
TILs [51]	Tissue-based	Moderate	Low	Low	Variable	Low
KIM-1 [62,63,64]	Circulating	Moderate–High	Moderate	Low	Moderate–High	Moderate
ctDNA [70,71]	Circulating	Emerging–Moderate	Low–Moderate	Low	Moderate–High	Emerging
NLR/SII [78]	Circulating	Moderate–High	Moderate–High	Moderate	Low–Moderate	Moderate
Integrated models [20,63,69]	Integrated	High	Moderate	Low	High	Moderate

High: supported by multiple large, independent studies or meta-analyses; Moderate: supported by several studies with some heterogeneity; Low: limited or inconsistent evidence; Emerging: preliminary data requiring further validation.

## Data Availability

No new data were created or analyzed in this study.

## Abbreviations

The following abbreviations are used in this manuscript:

ccRCC	clear cell renal cell carcinoma
BAP1	BRCA1-associated protein 1
PBRM1	polybromo 1
SETD2	SET domain containing 2
H3K36me3	trimethylation of lysine 36 on histone H3
VHL	von Hippel–Lindau
KDM5C	lysine demethylase 5C
mTOR	mechanistic target of rapamycin
TP53	tumor protein p53
PD-L1	programmed death-ligand 1
PD-1	programmed cell death protein 1
CD8	cluster of differentiation 8
KIM-1	kidney injury molecule-1
HAVCR1	hepatitis A virus cellular receptor 1
ctDNA	circulating tumor DNA
miRNA/miRNAs	microRNA/microRNAs
NLR	neutrophil-to-lymphocyte ratio
PLR	platelet-to-lymphocyte ratio
LMR	lymphocyte-to-monocyte ratio
SII	systemic immune-inflammation index
CRP	C-reactive protein
GPS	Glasgow Prognostic Score
VEGF-A	vascular endothelial growth factor A
IL-6	interleukin-6
CAIX	carbonic anhydrase IX
HIF	hypoxia-inducible factor
PI3K	phosphoinositide 3-kinase
AKT	protein kinase B
DDR	DNA damage response
IHC	immunohistochemistry
TILs	tumor-infiltrating lymphocytes
TLS	tertiary lymphoid structures
IMDC	International Metastatic Renal Cell Carcinoma Database Consortium
RFS	recurrence-free survival
DFS	disease-free survival
CSS	cancer-specific survival
OS	overall survival
PFS	progression-free survival
MRD	minimal residual disease
